# ChromoMap: an R package for interactive visualization of multi-omics data and annotation of chromosomes

**DOI:** 10.1186/s12859-021-04556-z

**Published:** 2022-01-11

**Authors:** Lakshay Anand, Carlos M. Rodriguez Lopez

**Affiliations:** Environmental Epigenetics and Genetics Group, Department of Horticulture, University of Kentucky, Lexington, KY 40546 USA

**Keywords:** Genome visualization, Multi-omics data visualization, R package

## Abstract

**Background:**

The recent advancements in high-throughput sequencing have resulted in the availability of annotated genomes, as well as of multi-omics data for many living organisms. This has increased the need for graphic tools that allow the concurrent visualization of genomes and feature-associated multi-omics data on single publication-ready plots.

**Results:**

We present chromoMap, an R package, developed for the construction of interactive visualizations of chromosomes/chromosomal regions, mapping of any chromosomal feature with known coordinates (i.e., protein coding genes, transposable elements, non-coding RNAs, microsatellites, etc.), and chromosomal regional characteristics (i.e. genomic feature density, gene expression, DNA methylation, chromatin modifications, etc.) of organisms with a genome assembly. ChromoMap can also integrate multi-omics data (genomics, transcriptomics and epigenomics) in relation to their occurrence across chromosomes. ChromoMap takes tab-delimited files (BED like) or alternatively R objects to specify the genomic co-ordinates of the chromosomes and elements to annotate. Rendered chromosomes are composed of continuous windows of a given range, which, on hover, display detailed information about the elements annotated within that range. By adjusting parameters of a single function, users can generate a variety of plots that can either be saved as static image or as HTML documents.

**Conclusions:**

ChromoMap’s flexibility allows for concurrent visualization of genomic data in each strand of a given chromosome, or of more than one homologous chromosome; allowing the comparison of multi-omic data between genotypes (e.g. species, varieties, etc.) or between homologous chromosomes of phased diploid/polyploid genomes. chromoMap is an extensive tool that can be potentially used in various bioinformatics analysis pipelines for genomic visualization of multi-omics data.

**Supplementary Information:**

The online version contains supplementary material available at 10.1186/s12859-021-04556-z.

## Background

Recent dramatic decrease in Next Generation Sequencing costs, and its application to the analysis of multiple levels of biological information (genomics, transcriptomics, epigenomics), has rapidly increased the number of multi-omics datasets available in public repositories. This has sifted the interest of researchers towards the annotation and comparison of such information at a genome or chromosome level. Besides identifying structural differences, comparison of genomic annotations can help understanding how variability within and between species affect phenotypes.

Currently, genomes are viewed using interactive web-based genome browser applications like JBrowse [1]. There is a paucity of non-web based independent tools capable of generating publication-ready interactive visualization of annotated genomes. This is more evident with the recent advent of diploid phased genomes of highly heterozygous samples [2]. The R [3] package *chromoMap*, is capable of annotating multiple genomic features, for the generation of interactive graphics. This allows the visualization in a single plot of chromosomes or chromosome sections of any living organism. The large genome size of certain species presents practical graphical challenges of displaying whole chromosomes within the available canvas space suitable for publications. ChromoMap, renders chromosomes as a continuous composition of windows, to surmount this restriction. Each window, consisting of a specific genomic range determined algorithmically based on chromosome length, displays information about the annotation in that region as a tooltip, allowing for plot interactivity.

## Implementation

**Fig. 1:**
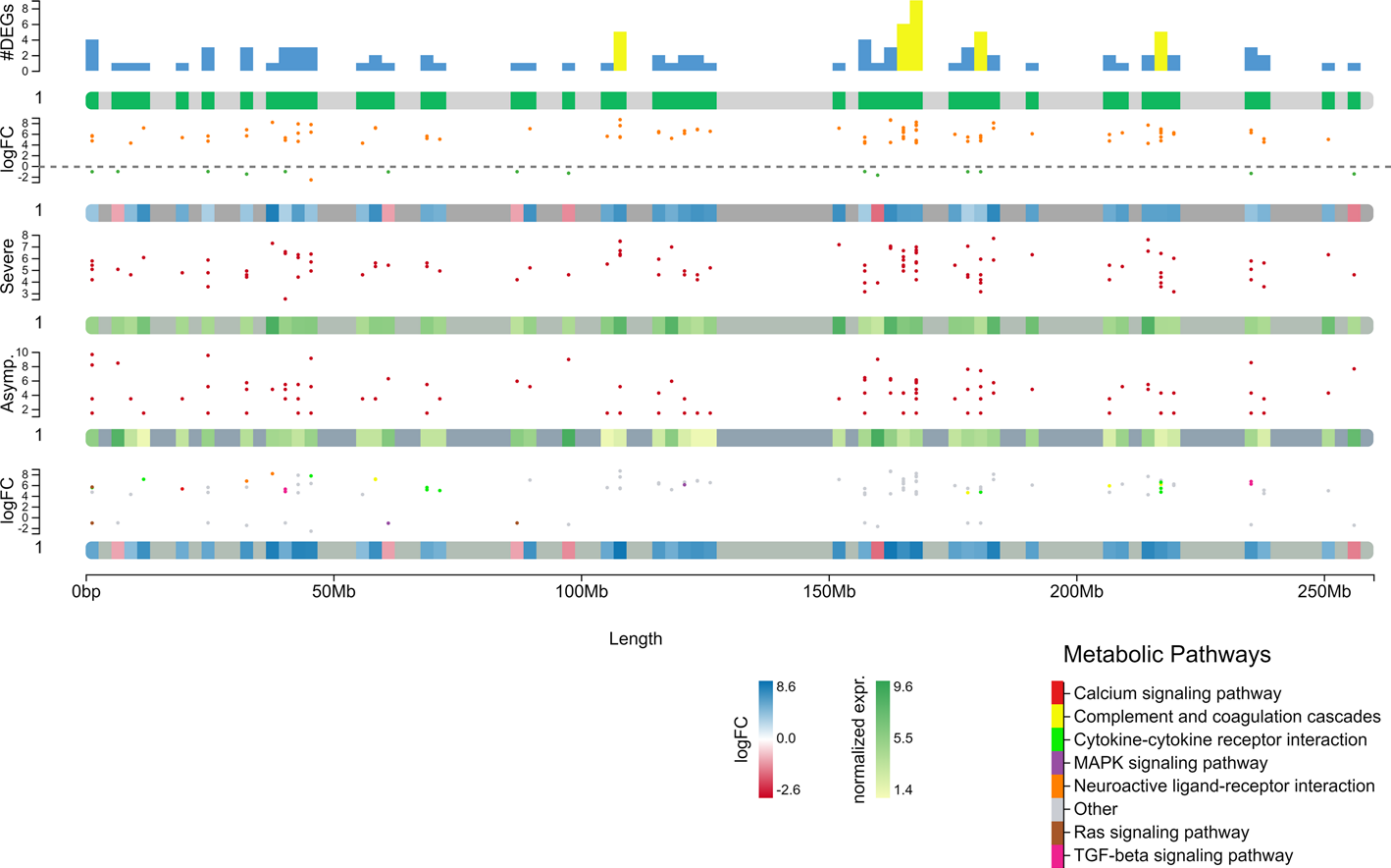
Example of chromoMap plot constructed using various features of chromoMap including polyploidy (used as multi-track), feature-associated data visualization (scatter and bar plots), chromosome heatmaps, data filters (color-coded scatter and bars). Differential gene expression in a cohort of patients positive for COVID19 and healthy individuals (NCBI Gene Expression Omnibus id: GSE162835) [12]. Each track contains information for chromosome 1 (For a complete interactive plot containing all 23 chromosomes see Additional file 1). Each track from top to bottom describes: (**1**) number of differentially expressed genes (DEGs) (FDR < 0.05) (bars over the chromosome depictions) per genomic window (green boxes within the chromosome). Windows containing ≥ 5 DEGs are shown in yellow. (**2**) DEGs (FDR < 0.05) between healthy individuals and patients positive for COVID19 visualized as a scatterplot above the chromosome depiction (genes with logFC ≥ 2 or logFC ≤ −2 are highlighted in orange). Dots above the grey dashed line represent upregulated genes in COVID19 positive patients. Heatmap within chromosome depictions indicates the average LogFC value per window. (**3–4**) Normalized expression of differentially expressed genes (scatterplot) and of each genomic window containing DEG (green scale heatmap) in (**3**) patients with severe/critical outcomes and (**4**) asymptomatic/mild outcome patients. (**5**) logFC of DEGs between healthy individuals and patients positive for COVID19 visualized as scatter plot color-coded based on the metabolic pathway each DEG belongs to


chromoMap is built using two languages, R and JavaScript. The R code, written using a procedural approach (as opposed to an object-oriented paradigm), handles the data input and data pre-processing part. The graphics rendering is entirely handled by the JavaScript part, constructed using vanilla JavaScript and D3 JavaScript library (version 4.0). The package was designed and tested using R version 4.0 and is compatible to run with R version 4.0 (or later) on multiple OS (Windows/macOS/Linux). R was chosen due its open-source nature as well as due to its general preference within the bioinformatics community. Annotated chromosomes are constructed by providing genomic co-ordinates, and optional secondary data, as either tab-delimited BED files or directly through R objects. A single R function is used for constructing a variety of different plots by changing its arguments values. For example, Fig. 1 was generated using the code below.

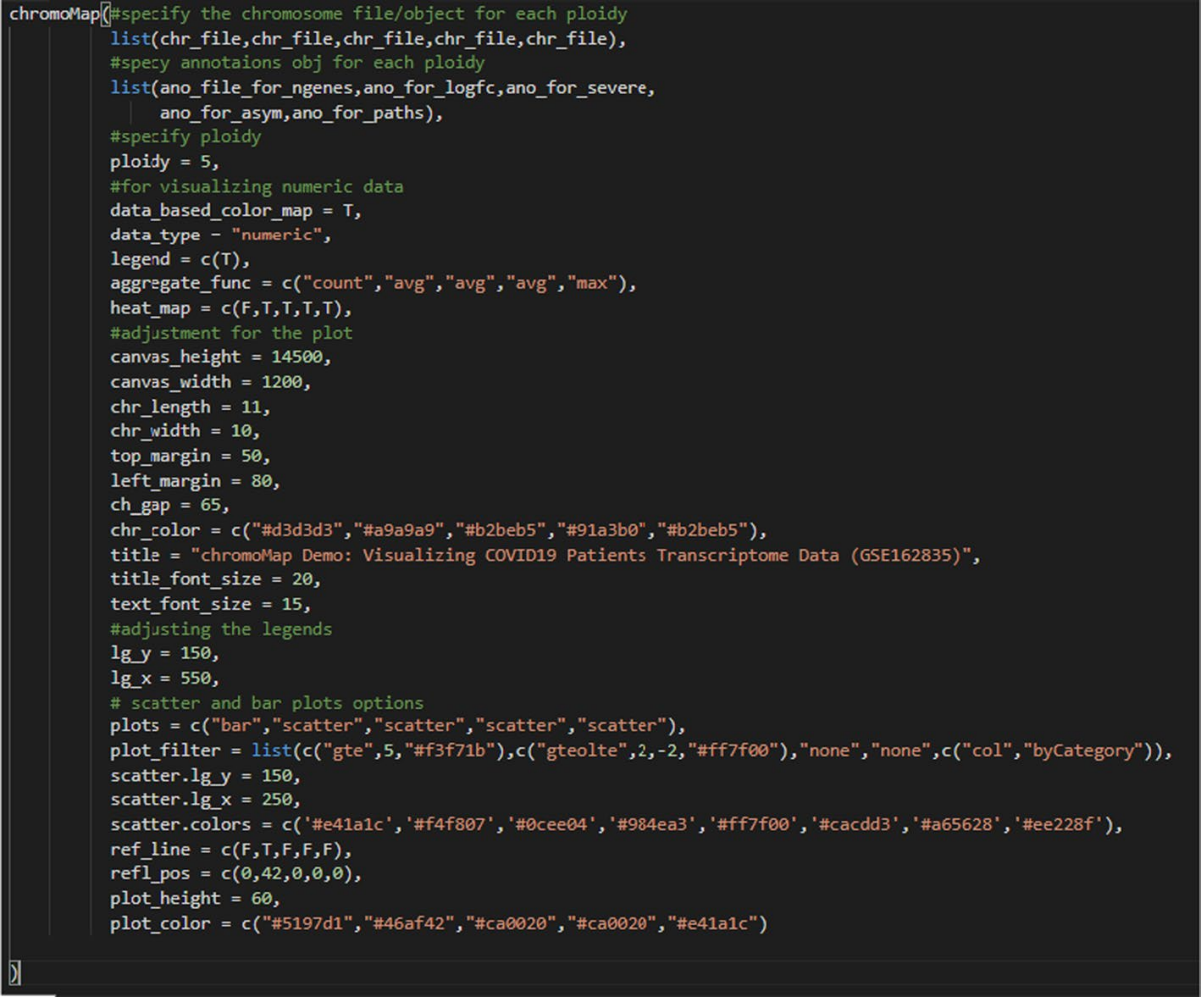


As shown in the example code above, the user inputs to the function fall into two categories: (1) the BED files or R objects, which specify the genomic coordinates of the chromosomes/chromosomal regions as well as the genomic coordinates for the studied genomic features, (genes, SNPs etc.), along with their associated data (e.g. gene expression values), and (2) the various ‘option’ arguments that are used to specify the graphic-properties of the plot, used for instance to turn a feature ON/OFF etc. Errors caused by the first type, such as user-provided files with incorrect structure or files created in an unsupported format, is handled in the implementation such that the program will first validate the file and will terminate the program with message on the console if the input file was incorrect. In addition to the detection of unsupported file format and/or structure, we implemented an out-of-bound annotations (i.e., annotation coordinates outside the boundary of the target chromosome or genomic feature) detection step. First, out-of-bound annotations are identified by the program and removed from the analysis. Then, the plot is rendered with the remaining annotations, together with an error message, displayed on the console to alert the user of the presence of such errors in their input files. The errors caused by the second type of inputs (e.g. the use of options not available for a given plot type, or failure to specify an option), are also handled in the implementation in a similar way (i.e., checking and exiting the program if encounter errors).

The generated plot, as viewed in RStudio’s viewer pane, offers users the possibility to zoom into a locus allowing them to explore their annotations of interest. Users can export the plot either as a static image [4–10], or as a stand-alone HTML file (see example in https://pouya-dini.github.io/equine-gene-db/#tab2) that contains an interactive plot that can be included in webpage accessible formats (e.g. Shiny Apps), as additional files in publications (See example Additional files 1 and 2), or embedded in RMarkdown documents.

## Features and applications

### Point and segment-annotations

**Fig. 2:**
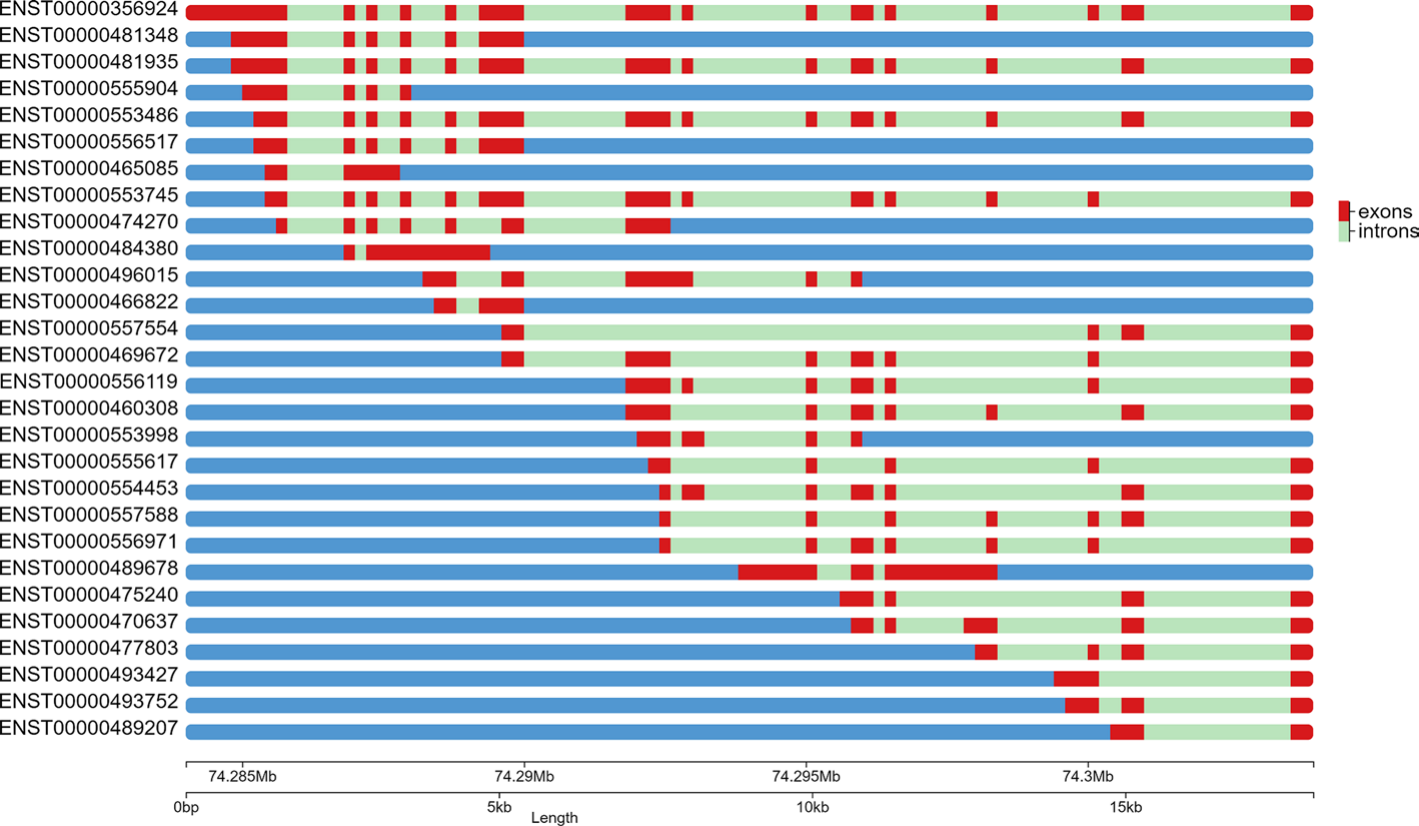
Example of chromoMap plot generated using the segment-annotation feature. 28 Splice variants of the human ABCD4 (ATP binding cassette subfamily D member 4) gene visualized using the segment-annotation feature. Exons are highlighted in red and introns in green. Feature coordinates were extracted from Ensembl BioMart [13]

ChromoMap provides the choice of two annotation algorithms, point-annotation and segment-annotation, differing in how annotations are visualized on the plot. Point-annotation ignores the element’s size, annotating it on a single base. Segment-annotation uses the element’s size to delimit its location. This can be advantageous when visualizing and annotating chromosomal regions or structural elements (Fig. 2).

### Group annotations

**Fig. 3:**
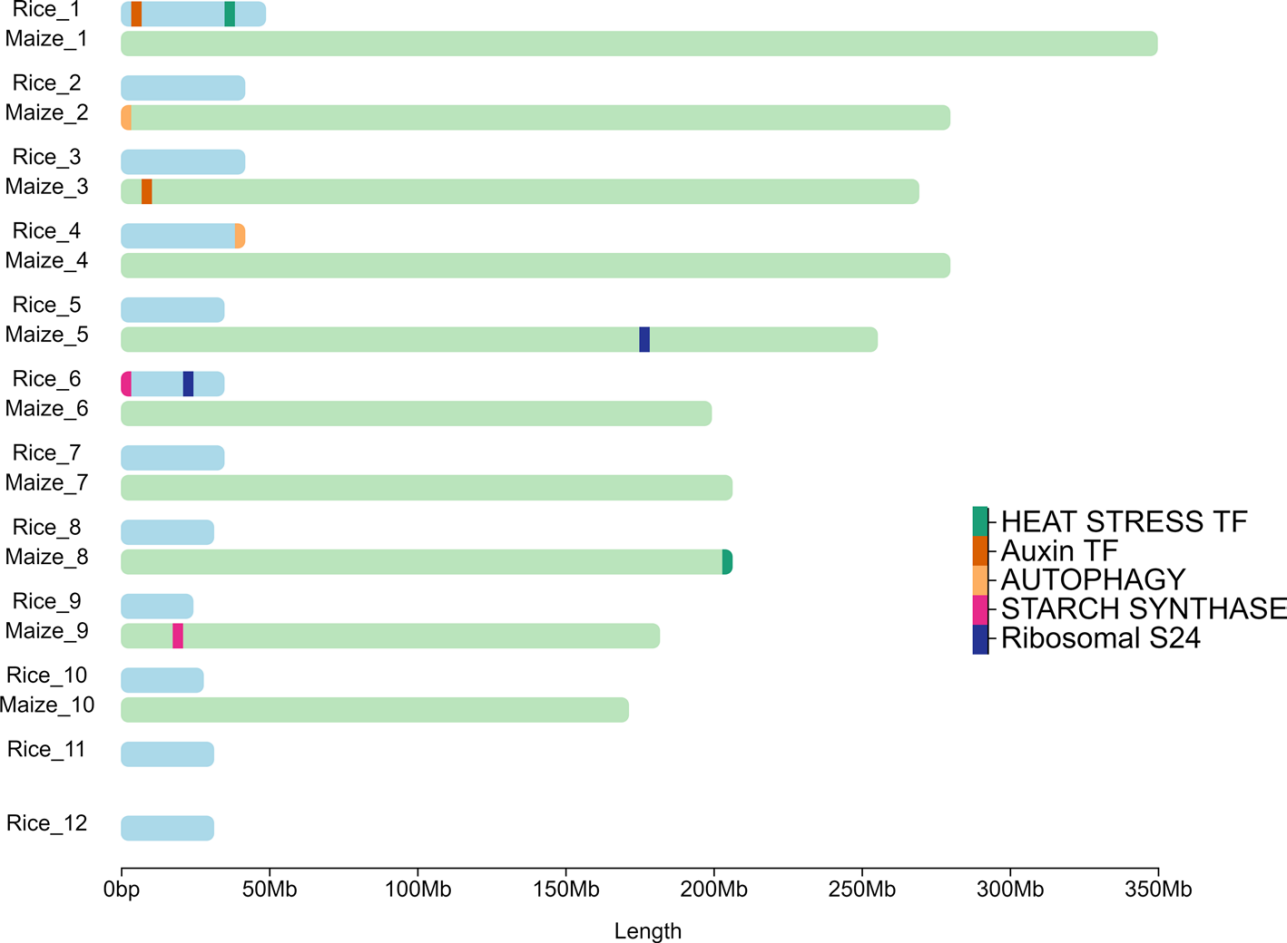
Example of chromoMap plot constructed using group annotations. Genomic location of 5 ortholog genes in the rice and maize genomes. Gene coordinates were extracted from Ensembl BioMart [13]

Visualizing annotations might require depicting annotations of groups of elements as opposed to individual ones. Groups can be color-coded for effective visualizations (Fig. 3).

### Hyperlinks and labelling

Allow the user viewing information embedded in tooltips associated to genomic features and to be redirected to preexisting webpages containing detailed information about such features. Additionally, users can use the ‘labelling’ option to display labels on static visualizations.

### Feature-associated data visualization

**Fig. 4:**
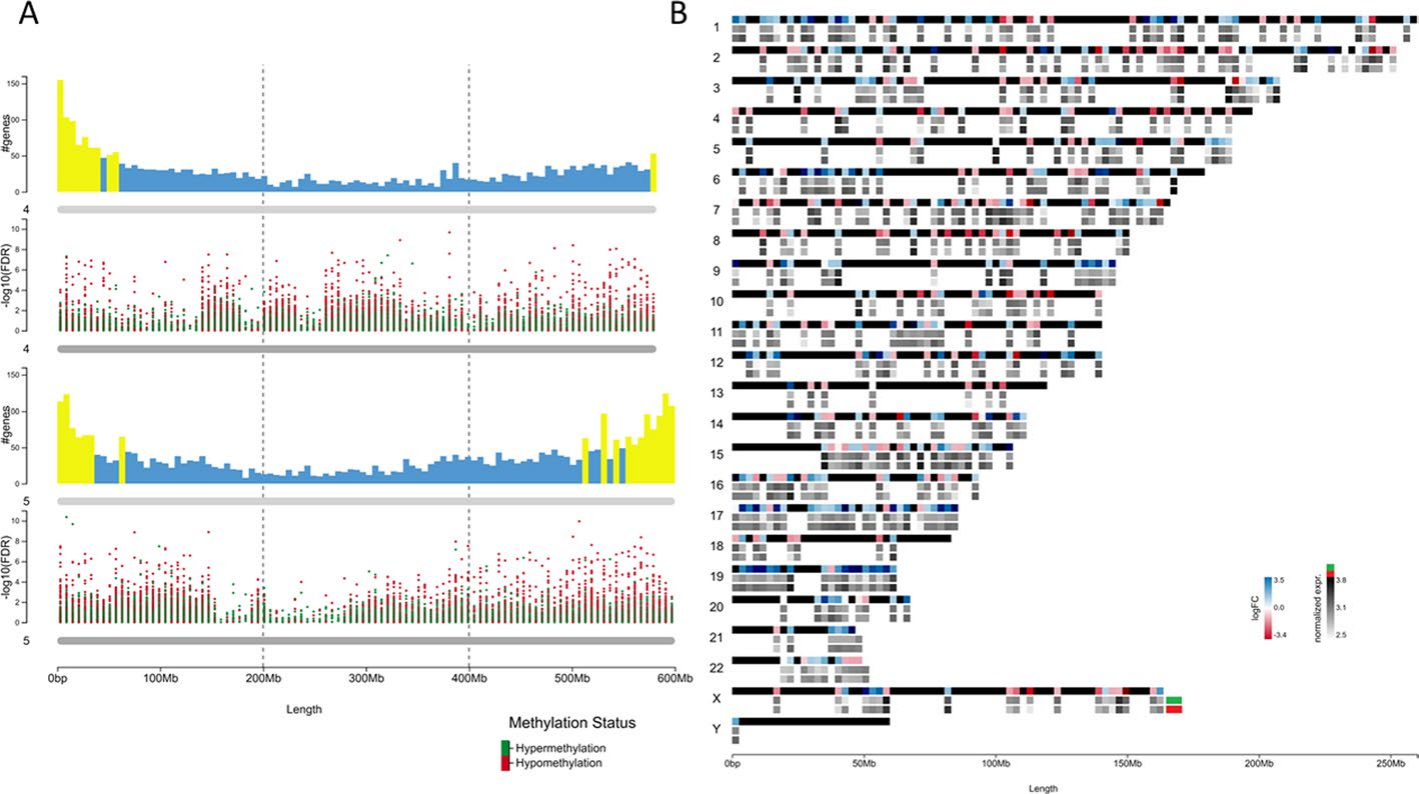
Feature-associated data visualization using chromoMap. **A** Visualization of genomic and epigenomic information for chromosomes 4 and 5 in barley (*Hordeum vulgare*). Vertical bars show the number of protein coding genes per genomic window (windows with ≥ 50 genes shown in yellow), and differential methylation (-Log10 (FDR)) of ms-GBS markers between plants grown under control conditions and under salt stress (75 mM NaCl) (Green: Hypermethylated markers under salt stress. Red: Hypomethylated markers under salt stress) European Nucleotide Archive (ENA) (Study Accession Number: PRJEB27251) [14]. **B** gene expression differences between healthy and breast cancer patients (NCBI Gene Expression Omnibus id: GSE59055) [15]. Solid black chromosomes depict difference in gene expression (logFC) between the healthy and cancer diagnose cohorts, while empty chromosomes depict the mean normalized expression for individuals within the control (Green) diseased (Red) cohorts

Feature-associated numeric data, such as gene expression, methylation status, copy number variants, feature density values, etc., can either be summarized as scatter/bar plots (Fig. 4a) or visualized as heatmaps (Fig. 4b). As each window represents a specific range of base pairs, multiple elements can be annotated within its range. ChromoMap uses aggregated data values (sum, average, min, max or count) for each window encompassing more than one element. Individual data values, for each element, can also be viewed in tooltips. Additionally, there is an ‘epi-tag’ feature that allows condition-based marking of loci. More advanced features include creating multidimensional scatter plots (Fig. 1-track 5) or applying mathematical filters on scatter/bar plots as depicted on Fig. 3 by Chidzanga et al. [11].

### Polyploidy (multitrack)

This feature allows rendering each chromosome set independently irrespectively of the species’ ploidy. This allows plotting chromosome sets that differ in size and number. This feature also allows the visualization of chromosome sets of different species for comparative genomic studies; or the visualization of highly heterozygous diploid genomes and homologous chromosomes pairs in phased diploid/polyploid genome assemblies (Fig. 1). This feature allows the visualization of biologically important variability that would be lost in a consensus sequence assembly.

### chromLinks

**Fig. 5:**
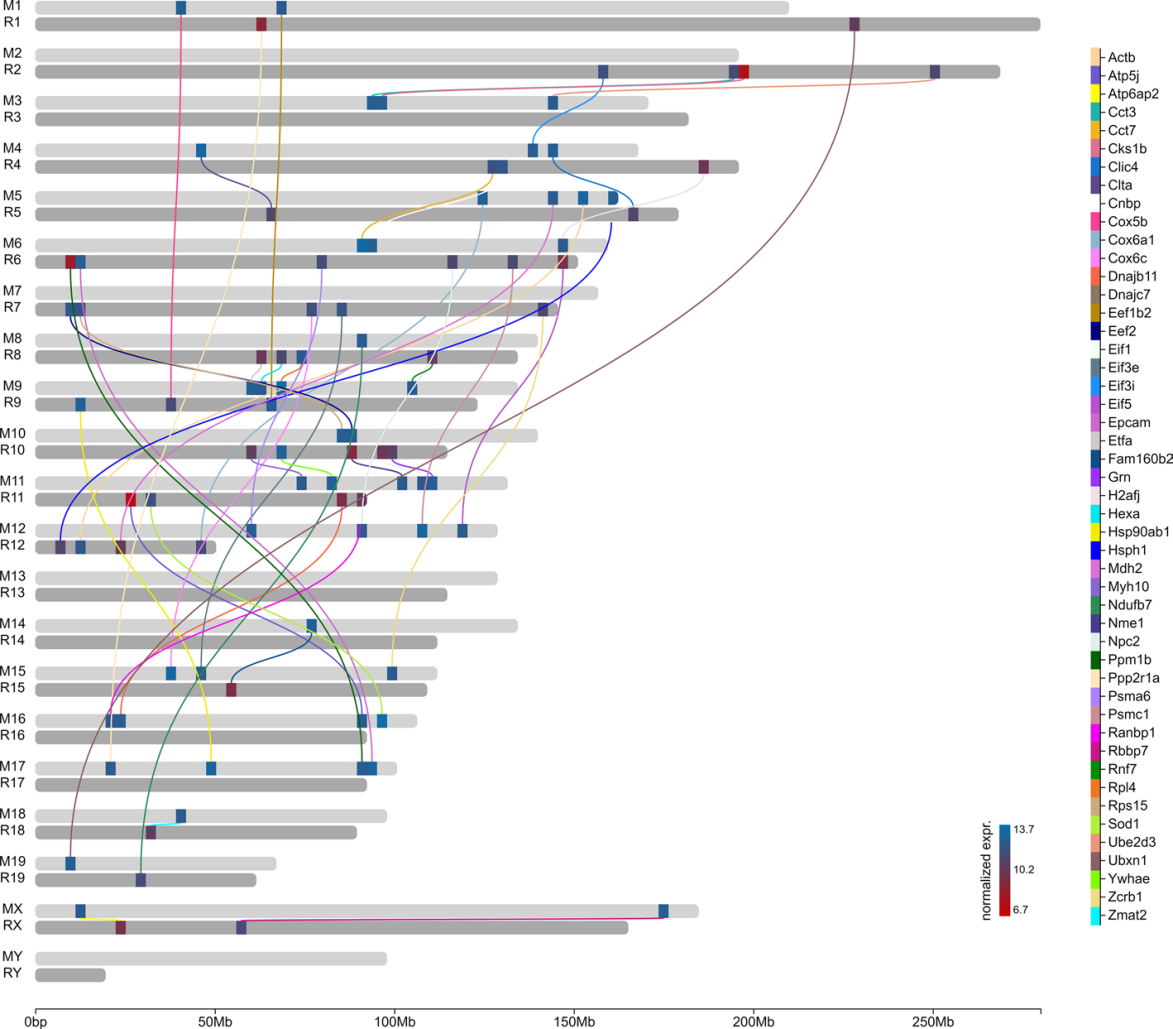
Example plot generated using chromLinks: Normalized expression of the top 50 highly expressed orthologous genes in mouse (*Mus musculus*) and rat (*Rattus norvegicus*) during pluripotent cell determination (NCBI Gene Expression Omnibus id: GSE42081) [16]. Orthologous gene pairs are connected with colored links. Each orthologous pair is indicated by a different link color. For an interactive version of this plot see Additional file 2

The linkage/correlation between any two annotated features can be visualized either as directed or undirected edges or as thick chord-like connectors. Moreover, data associated to these links can be visualized as color-coded links along with respective legends (Fig. 5). This feature can have several applications like for visualizing the homolog genes or to visualize co-expressed genes.

## Conclusion

ChromoMap is an efficient and user-friendly tool to visualise genomic elements, as well as its associated data (such as multi-omics data), in relation to their regional occurrence across chromosomes for any living organism with an available genome assembly. ChromoMap’s flexible plots permits the comparison of data uniquely associated to each strand of a given chromosome, and of homologous chromosome independently of their size. ChromoMap will allow the individual visualization of multi-omics data in all homologous chromosomes of phased diploid/polyploid genomes.

## Availability and requirements

ChromoMap is available under the GPL-3 Open Source license from: https://CRAN.R-project.org/package=chromoMap. A vignette is included for comprehensive description of its features. ChromoMap is also available as a containerized web application, referred as chromoMap App, that provides a GUI that will allow researchers to utilize all the above-mentioned features in an R-independent manner. The app can be run locally on the user’s computer. chromoMap App is available as a Docker Image at Docker Hub (https://hub.docker.com/r/lakshay57/chromomap-app). The datasets used and/or analyzed during the current study are available from the corresponding references.

Project name: chromoMap.

Project home page: https://lakshay-anand.github.io/chromoMap/index.html.

Operating system(s): Platform independent.

Programming language: R, JavaScript.

Other requirements: None.

License: GPL3.

Any restrictions to use by non-academics: license needed.

## Supplementary Information

**Additional file 1**. Example of chromoMap interactive plot constructed using various features of chromoMap including polyploidy (used as multi-track), feature-associated data visualization (scatter and bar plots), chromosome heatmaps, data filters (color-coded scatter and bars). Differential gene expression in a cohort of patients positive for COVID19 and healthy individuals (NCBI Gene Expression Omnibus id: GSE162835) [12]. Each set of five tracks labeled with the same chromosome ID (e.g. 1-22, X & Y) contains the following information: From top to bottom: (1) number of differentially expressed genes (DEGs) (FDR < 0.05) (bars over the chromosome depictions) per genomic window (green boxes within the chromosome). Windows containing ≥ 5 DEGs are shown in yellow. (2) DEGs (FDR < 0.05) between healthy individuals and patients positive for COVID19 visualized as a scatterplot above the chromosome depiction (genes with logFC ≥ 2 or logFC ≤ −2 are highlighted in orange). Dots above the grey dashed line represent upregulated genes in COVID19 positive patients. Heatmap within chromosome depictions indicates the average LogFC value per window. (3–4) Normalized expression of differentially expressed genes (scatterplot) and of each genomic window containing DEG (green scale heatmap) in (3) patients with severe/critical outcomes and (4) asymptomatic/mild outcome patients. (5) logFC of DEGs between healthy individuals and patients positive for COVID19 visualized as scatter plot color-coded based on the metabolic pathway each DEG belongs to.

**Additional file 2**. Example of interactive plot generated using chromLinks: Normalized expression of the top 50 highly expressed orthologous genes in mouse (Mus musculus) and rat (Rattus norvegicus) during pluripotent cell determination (NCBI Gene Expression Omnibus id: GSE42081) [16]. Orthologous gene pairs are connected with colored links. Each orthologous pair is indicated by a different link color.

## Data Availability

All datasets used to showcase chromoMap functionalities were previously published and are cited in the text. Datasets used to generate Figs. 1, 4b and 5 were obtained from the NCBI Gene Expression Omnibus, IDs: GSE162835 GSE59055, GSE42081 respectively. Data used for Fig. 4a was obtained from the European Nucleotide Archive: Study Accession No. PRJEB27251. Data used for Figs. 2 and 3 was obtained from Ensembl BioMart.

## Abbreviations

ms-GBS: Methylation sensitive genotyping by sequencing
DEG: Differentially expressed gene
BED: Browser extensible data
D3: Data-driven documents
CRAN: The comprehensive R archive network
